# Complications associated with removal of airway devices under deep anesthesia in children: an analysis of the Wake Up Safe database

**DOI:** 10.1186/s12871-022-01767-6

**Published:** 2022-07-15

**Authors:** Lisa Vitale, Briana Rodriguez, Anne Baetzel, Robert Christensen, Bishr Haydar

**Affiliations:** 1 Department of Pediatric Anesthesia, University of Michigan, Mott Hospital, 1540 E Hospital Dr SPC 4245, Ann Arbor, MI 48109-4245 USA; 2 4-917 Mott Hospital, 1540 E Hospital Dr SPC 4245, Ann Arbor, MI 48109-4245 USA; 3 US Anesthesia Partners Texas – South, P.O. Box 701090, San Antonio, TX 78270 USA; 4 4-951 Mott Hospital, 1540 E Hospital Dr SPC 4245, Ann Arbor, MI 48109-4245 USA; 5 4-914 Mott Hospital, 1540 E Hospital Dr SPC 4245, Ann Arbor, MI 48109-4245 USA; 6 4-911 Mott Hospital, 1540 E Hospital Dr SPC 4245, Ann Arbor, MI 48109-4245 USA

**Keywords:** Airway extubation, Airway management, Anesthesia, General, Complications, Arrest, Cardiopulmonary, Pediatrics

## Abstract

**Background:**

Previous studies examining removal of endotracheal tubes and supraglottic devices under deep anesthesia were underpowered to identify rare complications. This study sought to report all adverse events associated with this practice found in a large national database of pediatric anesthesia adverse events.

**Methods:**

An extract of an adverse events database created by the Wake Up Safe database, a multi-institutional pediatric anesthesia quality improvement initiative, was performed for this study. It was screened to identify anesthetics with variables indicating removal of airway devices under deep anesthesia. Three anesthesiologists screened the data to identify events where this practice possibly contributed to the event. Event data was extracted and collated.

**Results:**

One hundred two events met screening criteria and 66 met inclusion criteria. Two cardiac etiology events were identified, one of which resulted in the patient’s demise. The remaining 97% of events were respiratory in nature (64 events), including airway obstruction, laryngospasm, bronchospasm and aspiration. Some respiratory events consisted of multiple distinct events in series. Nineteen respiratory events resulted in cardiac arrest (29.7%) of which 15 (78.9%) were deemed preventable by local anesthesiologists performing independent review. Respiratory events resulted in intensive care unit admission (37.5%), prolonged intubation and temporary neurologic injury but no permanent harm. Provider and patient factors were root causes in most events. Upon investigation, areas for improvement identified included improving patient selection, ensuring monitoring, availability of intravenous access, and access to emergency drugs and equipment until emergence.

**Conclusions:**

Serious adverse events have been associated with this practice, but no respiratory events were associated with long-term harm.

## Background

Removal of endotracheal tubes and supraglottic airway devices under deep anesthesia [1, 2] is performed for provider preference to reduce coughing and airway activation or to potentially improve operating room efficiency. This is often accompanied by mild airway obstruction [3]. In adults, this has been associated with a 13% incidence of airway complications [4]. In children, adverse events resulting from removal of airway devices under deep anesthesia have been evaluated in small, prospective studies, and the largest meta-analysis techniques amassed 1395 patients who underwent removal of a supraglottic airway device under deep anesthesia [3]. None of these have identified serious adverse events such as cardiac arrest [5], which is not unexpected as the rate of anesthesia-related cardiac arrest in pediatric anesthesia is only 3.3 per 10,000 [6]. There are previous reports of serious complications occurring with deep extubation [7, 8], but only one definitively identifying cardiac arrest that was associated with deep extubation [8]. A cardiac arrest focused registry did identify that four of six laryngospasms leading to cardiac arrest were associated with removal of airway devices while the patient was “partially but not fully awake” [9]. There have been no specific evaluations of rare but serious adverse events that result from this practice.

National registries of adverse events are ideal for identifying such events. Wake Up Safe, a multi-institutional pediatric anesthesia patient safety organization and quality improvement initiative, created one such registry [10]. This database also contains detailed root cause analysis of each included adverse event.

The primary objective of this study was to create a list of adverse events associated with removal of either endotracheal tubes or supraglottic devices under deep anesthesia and to identify any associated patient harm. A secondary objective was to identify the root causes of these events and report learning points identified from those root cause analyses.

## Methods

Wake Up Safe, a pediatric anesthesia patient safety organization, created and maintains a registry of adverse events which includes demographic and event data, also known as the Wake Up Safe database. The registry’s creation and data entry are described below. Each adverse event was prospectively defined using expert consensus prior to the launch of the database [11]. Adverse events selected for possible inclusion undergo local review by a panel of anesthesiologists trained in root cause analysis who determine the root causes and outcome of the event. They perform a review of the medical record, conduct interviews with staff who were involved in or witnessed the event, and conduct additional investigations as needed. This may include, for example, a discussion with the manufacturer or supplier of medical equipment involved in an adverse event. They assess whether the event was preventable and describe learning potential from the event. Events are then entered in the registry in a de-identified fashion [10]. Detailed descriptions of the database and database extract preparation for research purposes have been previously described [11]. Institutional Review Board approval for participation in this registry was obtained at all member sites, which deemed individual patient consent not required as all data are de-identified prior to upload to the database. The University of Michigan Institutional Review Board deemed the use of this database as meeting criteria for exemption from additional review. The University of Michigan Research Ethics and Compliance Committee deemed that this research was exempt and required no informed consent, as it was an analysis of a de-identified dataset.

For this study, inclusion criteria were as follows: any adverse events that occurred during the emergence phase or early recovery phase of general anesthesia, where the endotracheal tube or supraglottic airway was intentionally removed in an anesthetized state, according to database variables or to the narrative data included in each event. Events were screened using database variables that identified anesthetic type and timing of the adverse event. Events using general anesthesia and events that occurred from removal of the airway device until 2 h after post anesthesia care unit (PACU) arrival were screened in for potential inclusion. Next, a panel of 3 anesthesiologists (Anthony Franchetti, BH and BR) reviewed all screened events to independently assess whether removal of the airway device under deep anesthesia potentially caused, contributed to, or worsened the severity of the adverse event. For example, cardiac events were included if hypoxemia, hypercarbia, and/or hypotension (per local event review) likely caused or contributed to the adverse event. Consensus on inclusion criteria was required for events to be included in this study.

We extracted event data and variables using the same methodology as previous studies involving the Wake Up Safe database [11]. The etiology for respiratory events was determined from the event narrative field and specific database variables. Cardiac arrest was defined by use of chest compressions, Pediatric Advanced Life Support-defined epinephrine dosing for cardiac arrest [12], or the use of a cardiac arrest template for the reported event. A “learning points” variable is contained in each event in the database. These data were extracted, along with any additional learning points contained within the event narrative, as defined by a panel of study authors. Consensus on the learning points in events was achieved through direct discussion of study authors. We adhered to STROBE guidelines for the entirety of this project [13].

## Results

A Wake Up Safe database extract from 6/6/2019 was obtained and contained 3652 events. The initial screen identified 102 candidate events. One duplicate event was removed and sixty-six events were confirmed as meeting inclusion criteria. Events were excluded for the following reasons: inability to establish the depth of anesthesia during airway device removal; airway device was unintentionally removed; or that the event could not be related to airway device removal. Included events consisted of two events with a primary cardiac etiology (3.0% of all events) and sixty-four respiratory events (97.0%). The cardiac events consisted of a transient supraventricular tachycardia in an otherwise healthy 2-year-old male, and a cardiac arrest in a 15-year-old male resulting in extracorporeal membrane oxygenator cannulation and death, subsequently found to be related to a pulmonary embolus. Both of these were deemed as related to patient disease and unrelated to anesthesia care.

Respiratory events are summarized in Table 1, with some events consisting of multiple distinct events in sequence, such as laryngospasm followed by aspiration. Respiratory events involved patients aged 7 weeks to 19 years, with a median age of 22 months. Patient weight ranged from 3.25 to 76 kg. Most were rated ASA-PS 1 or 2 (40, 62.5%) and presented for operative procedures (48, 75.0%). The most common types of procedures included otolaryngological (15, 31.3%) and urogenital (12, 25.0%). Airway devices used in these events included supraglottic airways in twenty-six events (40.6%), endotracheal tubes in eight events (12.5%), and the remainder were not specified. Timing of adverse events varied. Forty-six events (71.9%) were listed as occurring while still in the anesthetizing location, with the remainder listed as occurring in PACU. At least eleven events were associated with transport, with six occurring during transfer from the operating room table, and five occurring during transport to PACU or on PACU arrival. PACU events included bronchospasm, laryngospasm, and respiratory failure. Two events consisted of hypoxic and/or hypercarbic respiratory failure which took several hours to be recognized. Laryngospasm in PACU was delayed up to 40 min after removal of an airway device under deep anesthesia.

**Table 1:** Respiratory events

Etiology	Contributing Factors
Laryngospasm 35 (54.7%)	Anesthesia 53 (86.9%)
Airway obstruction 7 (10.9%)	Patient disease 39 (63.9%)
Emesis 5 (7.8%)	Perioperative team 4 (6.6%)
Apnea 4 (6.3%)	Surgical issues 3 (4.9%)
Bronchospasm 4 (6.3%)	Other 3 (4.9%)
Other/Not Specified 13 (20.3%)	
Multiple events 7 (10.9%)	

Some patients had more than one etiology. “Other” includes equipment issues and verbal miscommunication. “Location” refers to where the event occurred, which may not have been the anesthetizing location. Contributing factors were assessed in 61 of 64 events (95.3%)

Twenty-four respiratory events resulted in intensive care unit admission as a result of the event (37.5%). Nineteen respiratory events progressed to cardiac arrest (29.7%). Of these, fifteen were deemed as preventable (78.9%). Fourteen cardiac arrests resulted in no harm, while five events (26.3% of arrests; 7.8% of all respiratory events) resulted in temporary harm which included reintubation and ICU admission for monitoring. Two patients took weeks to recover. A 2-year-old with VACTERL association had laryngospasm then vomiting resulting in aspiration pneumonitis requiring high-frequency oscillatory ventilation. The patient also developed shock and pulmonary hypertension and was extubated after ten days. A 4-year-old became hypoxemic in PACU, received chest compressions and was reintubated. Several days later they were extubated and noted to have new ataxia and speech difficulties which resolved by hospital discharge one week later.

Root cause analysis included patient disease or a provider factor as a primary or secondary cause in all events. Provider factors most frequently identified included judgment, technical errors, inexperience, and cognitive biases. Equipment factors were identified in two events and a verbal miscommunication was a factor in one event. Only one event was associated with a handover. Events were typically preventable (30/47, 63.8%) and had learning potential (38/49, 77.6%) during local review and root cause analysis. All learning points listed within the Wake Up Safe database entries for these events are included in Table 2 and represent countermeasures specific to individual adverse events that occurred in this case series.

**Table 2:** Learning points from deep extubation-associated respiratory events

Increased awareness of deep extubation-associated events is needed
Opioids should be administered with caution, especially in patients at elevated risk for apnea and airway obstruction
Patients should have reliable intravenous access prior to deep extubation
In off-site locations, support staff may not know how to assist with respiratory adverse events. Deep extubation in off-site locations should be approached with caution
Attending anesthesiologists should be present with the patient during transport after deep extubation
Providers skilled at managing airway obstruction and laryngospasm should remain with the patient until emergence from anesthesia
Medications for treatment of laryngospasm should be immediately available until emergence from anesthesia
Deep extubation should be approached with caution in patients with airway abnormalities such as micrognathia, or in syndromes that may be associated with difficult airway
Close monitoring during transport and in PACU following deep extubation is essential. Consider capnography if available
Drugs and equipment for treatment of airway obstruction and laryngospasm should accompany the patient during prolonged transport, such as between floors
Patients may appear to remain deeply sedated following deep extubation as a result of hypercapnic respiratory failure. Prolonged emergence should prompt for further evaluation
Airway obstruction associated with deep extubation may result in post-obstructive pulmonary edema
Emergency equipment such as “Anesthesia Help” or “Code Blue” buttons should be tested regularly, and emergency carts in PACU should be stocked appropriately

*PACU* Post-anesthesia care unit. These learning points are compiled from entries within the Wake Up Safe database and also apply to removal of supraglottic devices under deep anesthesia

## Discussion

This is the largest series of serious adverse events relating to removal of airway devices under deep anesthesia, some of which have led to cardiac arrest and even death. The overwhelming majority of reported events were respiratory in nature which resulted in no lasting harm. There were few systems-related root causes and reviewers frequently noted that anesthesia provider decision-making largely contributed to these preventable serious adverse events. Event reviewers at local Wake Up Safe member sites who performed the root cause analyses clearly noted that basic education on this practice is needed; learning points are collected in Table 2. While some may seem obvious, this reflects a common retrospective cognitive bias [14] and readers may choose to compare the practice at their own institutions against these suggestions.

Further study is needed to develop consensus on risk factors for adverse events associated with deep extubation. Readiness assessment for awake extubation has only been recently formally investigated [15], and experts continue to disagree on criteria for deep extubation [1, 2]. Deep extubation remains controversial [1] and is less commonly performed than awake extubation [16]. For supraglottic devices, removal under deep anesthesia appears to be associated with fewer adverse events except for minor airway obstruction [3], though these studies generally do not constitute high-quality evidence [17]. In high-risk tonsillectomy patients, the benefit may be limited to reduced coughing and desaturation in PACU [18]. Caudal anesthesia appears to reduce the anesthetic dose required for deep extubation of supraglottic devices [18]. For endotracheal tubes, dexmedetomidine appears to reduce the inhaled anesthetic dosing requirement for deep extubation [5, 19, 20]. In adult patients, remifentanil demonstrated similar effects [21]. Though positioning patients laterally may help prevent adverse events [22, 23], patient movement and position changes under inadequate anesthesia may provoke adverse events [11].

This study included nineteen preventable cardiac arrests. While perioperative cardiac arrest is a thankfully rare event [5], further reductions can be achieved through changes in the systems of care [24]. This would help achieve the goal of Wake Up Safe: to reduce perioperative adverse events [8]. Table 2 lists countermeasures that could be implemented into local protocols or guidelines in an effort to prevent these events. These may include maintenance of intravenous access, staff, equipment, and medications near the patient until emergence from anesthesia has occurred. Cognitive aids for extubation have been described in the ICU literature [25, 26], but an opportunity exists to create and study these in the intraoperative setting especially in relation to deep extubation.

This study may be limited by missed events, selection bias, and limitations of the de-identified dataset, similar to previous Wake Up Safe reports [11]. Our methodology precluded calculation of the incidence of these events or further analyzing patient risk for adverse events. It was not possible to confirm to what degree removal of the airway device under deep anesthesia contributed to these adverse events. Due to limitations in the database, we were unable to ascertain what criteria were used to judge the patient’s depth of anesthesia at airway device removal. We were also unable to confirm the type of airway device used in some cases. Another limitation is that additional details about the surgical procedure or anesthetic technique were not included in this study. For simplicity, we elected to handle primary and secondary root causes similarly. We were unable to study other potential consequences of these complications, including increased time, expense or resource utilization, due to the limitations of the database. Some of these events may have been previously reported in other analyses of the Wake Up Safe database.

## Conclusions

Most adverse events related to removal of airway devices under deep anesthesia have been respiratory in nature without long-term sequelae. Most events are preventable. Adverse event reviewers suggested that these events may be prevented or mitigated through specific countermeasures. These include maintenance of intravenous access, staff with airway expertise, and equipment and emergency medications at the patient bedside until emergence from anesthesia.

## Data Availability

The data that support the findings of this study are available from Wake Up Safe but restrictions apply to the availability of these data, which were used under license for the current study, and so are not publicly available. Data are however available from the authors upon reasonable request and with permission of Wake Up Safe by contacting corresponding author LV.

## Abbreviations

PACU: Post-anesthesia care unit
ICU: Intensive care unit
VACTERL: Vertebral abnormalities, anal atresia, cardiac defects, tracheo-esophageal fistula, renal abnormalities, and limb abnormalities
ASA-PS: American Society of Anesthesiologists physical status
